# Genome-Wide Identification of P450 Genes in Chironomid *Propsilocerus akamusi* Reveals Candidate Genes Involved in Gut Microbiota-Mediated Detoxification of Chlorpyrifos

**DOI:** 10.3390/insects13090765

**Published:** 2022-08-24

**Authors:** Zeyang Sun, Yue Liu, Haixuan Xu, Chuncai Yan

**Affiliations:** College of Life Sciences, Tianjin Key Laboratory of Conservation and Utilization of Animal Diversity, Tianjin Normal University, Tianjin 300387, China

**Keywords:** *Propsilocerus akamusi*, P450 gene, gut microbiota deficient, chlorpyrifos, regulation

## Abstract

**Simple Summary:**

*Propsilocerus akamusi* is considered as a pollution-tolerant chironomid species in aquatic ecosystems. The capability of adapting to various contamination has been proved to rely on certain detoxifying enzymes, such as cytochrome P450 (P450), glutathione S-transferase (GST), etc. In the present study, 64 P450 genes were derived and characterized based on the available genome sequence. In addition, there is a potential role of gut microbial commensals in regulating local P450s when individuals have to deal with environmental stressors. Two screened P450s of gut tissue, PaCYP3998B1 and PaCYP3987D1, were remarkably decreased in chlorpyrifos-challenged subjects with deficient gut microbiota; this result suggested a possible function of gut microbial communities in enhancing host endogenous detoxification capability.

**Abstract:**

Chironomids commonly dominate macroinvertebrate assemblages in aquatic habitats and these non-biting midges are known for their ability to tolerate contaminants. Studies regarding the interplay between gut microbiota and host detoxification ability is currently a point of interest. Cytochrome P450s (P450s) are critical metabolic enzymes in which a subset is involved in xenobiotic detoxification. In this study, we first conducted an integrated global investigation of P450s based on the whole genomic sequence of *Propsilocerus akamusi* and retrieved a series of 64 P450 genes which were further classified into 4 clans and 25 families on the basis of phylogenetic relationships. With assistance of RNA-Seq and RT-qPCR validation, the expression profile of screened PaP450s in guts was compared between chlorpyrifos-challenged larvae with deficient gut microbiota (GD) and those with a conventional gut community (CV). An increasing prevalence of chlorpyrifos from sublethal to lethal dosages induced a greater mortality rate of individuals coupled with remarkable downregulation of 14 P450s in GD larval guts when compared to CV ones. Moreover, it turned out that the decreased level of PaCYP3998B1 and PaCYP3987D1 might imply impaired host endogenous detoxification capability potentiated by gut dysbiosis, reflected by a remarkably severe mortality in GD larvae treated with lethal chlorpyrifos. Collectively, our study unveiled candidate P450 genes that might be mediated by gut symbionts in chlorpyrifos-challenged *P. akamusi* larvae, possibly facilitating further understanding of the detoxified mechanism that chironomids might employ to alleviate poisonousness.

## 1. Introduction

Cytochrome P450s monooxygenases (CYPs or P450s) are known as one of the largest enzymatic protein superfamilies partially due to the occurrence of gene duplication and are virtually distributed in all kingdoms of organisms [1]. The critical importance of P450s has been emphasized by their interactions with endogenous and exogenous compounds. The monooxygenase function performed by P450s catalyzes the conjunction of polar hydroxyl groups to the substrate and causes the targeted substrates to become more reactive and hydrophilic for excretion. The sequence homology enables P450s to be categorized into bacterial and eukaryotic classes, among which annotated enzymes would be assigned with a family number (e.g., CYP1) followed by a subfamily letter (e.g., CYP1A). A particular number for a distinct isoform will then be appended for further differentiation (e.g., CYP1A6).

It has long been recognized that insects are deemed as evolutionally successful metazoans because of their specialized adaptation to diverse ecological niches, even if some are life-threatening or challenging. Insects have evolved a sophisticated system to protect themselves and, specifically, they are able to utilize a rich pool of genes as biological defenses against detrimental toxins from surrounding habits or other sources [2]. It has been widely acknowledged that insect cytochrome P450s normally act as detoxifying enzymes when individuals confront environmental stressors, such as pesticides and phytochemicals [3,4]. Over-production or constitutive induction of multiple P450s has been verified as the protective strategy that help hosts confer pesticide detoxification via biotransformation or biodegradation [5,6]. For example, an excessive amount of CYP6P3 in *Anophele gambiae* discloses a cross-resistance to pyrethroids and carbamate [7] and an upregulation of CYP4C71 in *Laodelphax striatellus* is bestowed with the ability to metabolize imidacloprid [8]. Likewise, the reversal of insecticide tolerance might result from downregulation of P450s. For instance, RNAi-based knockdown of CYP9A10 could increase the mortality rate of xanthotoxin-exposed beet armyworm [9] and the depressed CYP6BG1 renders diamondback moth more susceptible to permethrin [10]. Previous research has claimed that the P450-mediated resistance of insects to synthetic agrochemicals could be attributed to multiple copies of P450 genes [11], transcription regulation [12], changes in amino acid sequences [13], and other molecular regulation modes. Moreover, insect P450s have also been implicated with imperative roles in fundamental physiological processes, such as the biosynthesis of hormones and pheromones during juvenile and molting stages [14].

In recent decades, the genomic sequencing rush has allowed the determination and analysis of an ever-growing explosion of P450 members in diverse insect species. According to a plethora of investigations, the number of P450s, functional relevance, expression pattern, and phylogenetic relationship considerably vary among discrepant insect species. Quite a large number of P450s have been curated from certain species; for example, there are 204, 143, and 112 P450s discovered in *Culex quinquefasciatus* [15], *T. castaneum* [16], and *Anopheles sinensis* [5], respectively. Meanwhile, only 37 P450s are documented in *Pediculus humans* [17], which is a relatively small subset. Four prominent clusters called clans are introduced to further categorize insect P450s on the basis of sequence similarities and these four clades present as Clan 2, Clan 3, Clan 4, and Mitochondria (Mito) Clan. The evolutionarily functional divergence of abundant insect P450s might, in turn, assist the unrivaled diversity of insects and their world domination status.

Chironomids, the mosquito-resembled non-biting midges, are widely distributed in freshwater ecosystems and exhibit predominance among benthic invertebrates. A wide array of contaminants produced by industries as well as agricultural activities have been detected in surface water and suspended sediments. These toxic materials could be bio-accumulated in chironomid larvae and then pass along the food chain since chironomids are critical food sources for amphibians, fish, and some predatory insects. It is worth noting that some representative taxa display generalized occurrence, which highly relies on their acclimation to adverse habitats, such as eutrophication [18] or repository contaminants from anthropogenic disturbance (e.g., heavy metals or organic compounds). However, only a limited number of studies have reported protective mechanisms that chironomids utilize to cope with stressors. A suite of genes involved in the detoxification system of *Chironomus riparius* are elevated after treatment with xenobiotics, indicating a potential role in alleviating poisonousness [19]. The bacterial consortium of *Chironomus transvaalensis* and *C. riparius* could protect hosts by attenuating harmful effects of invading heavy metals [20]. To date, research pertinent to the adaptive and defensive tactics of chironomids to defend against potential toxins is still the tip of the iceberg and requires further investigation.

The alimentary canal is the insect’s primary site of environmental toxins and could act as the first line of defense. For example, a series of P450s are detected in gut tissues of insects and aid in defending chemical warfare [21]. Moreover, symbiotic microbes populating the digestive tracts of insects have been increasingly demonstrated to have roles in limiting toxic effects and maintaining fitness when hosts must cope with environmental stressors [22]. Bacterially facilitated tolerance of pesticides has been reported in some insect species since some microorganisms show superior potency in direct degradation of exogenous compounds [23]. More importantly, scientists are waking up to an accepted viewpoint that the presence of a balanced gut microbiota could also maintain host health via an indirect regulation of detoxification-related metabolism. For instance, the partial removal of bacterial communities in mosquitos could turn temephos-resistant strains into susceptible ones due to the dramatic activity reduction of detoxified enzymes, such as GSTs [24]. A severe mortality rate of gut microbiota-deficient honeybees was observed when samples were subjected to pesticides, probably resulting from the suppression of certain P450s in gut tissues [25].

*Propsilocerus akamusi* (Tokunaga, 1938) is an abundantly dominant species in Eastern Asia and rearing individuals in a laboratory condition is undemanding [26]. Chlorpyrifos (CPF), a globally applied organophosphate insecticide for agricultural activities, usually runs off to surface drainage and continuously raises environmental concerns for aquatic ecosystems. This broad-spectrum insecticide could damage the nervous system of targeted organisms by irreversibly promoting the accumulation of acetylcholine and the dysfunctional nerve increases the death rate [27]. It is interesting to notice a phenomenon where the lethal concentration of CPF for the 4^th^ larvae of *P. akamusi* could reach 68 μg/L (96-h LC_50_, Supplementary Table S1), which is higher than that for other chironomid species [28]. This feature makes *P. akamusi* an excellent candidate to evaluate the subsequently emerged detoxification process.

In this paper, the P450 multigene family of *P. akamusi* was firstly characterized and analyzed with the availability of the chromosome-assembled genome sequence. We then established a gut microbiota-deficient model of *P. akamusi* with the support of antibiotics for the first time and attempted to reveal a possible interaction between bacterial colonizers and the regulation of detoxification-associated genes, particularly P450s, in gut tissues of larvae treated with both a lethal and a sublethal dosage of CPF. RNA-Seq together with quantitative real time PCR (RT-qPCR) technology were employed to compare the expression profile of candidate P450s between normal guts and microbiota-reduced guts in CPF-administered larvae. Our study was committed to providing general information on P450 genes in *P. akamusi* as well as their responses to CPF in digestive tracts with partially obliterated microbial commensals. We attempted to explore a latent association between gut microbiota and detoxified P450s in *P. akamusi*, allowing investigators to continue follow-up toxicological studies and elucidate feasible strategies for chironomids to combat detrimental factors.

## 2. Methods and Materials

### 2.1. Sequence Retrieval of P450s in P. akamusi

The availability of the *P. akamusi* genome sequence and annotation files (Bioproject accession: PRJNA698390) were used to obtain the whole-genome protein sequences. P450 amino acid sequences of *Drosophila melanogaster* were downloaded from the Cytochrome P450 homepage (http://drnelson.uthsc.edu/CytochromeP450.html, accessed on 15 December 2021) and then served as the query for screening the transcriptome databases of *P. akamusi*. P450 homologs of *P. akamusi* were initially deduced based on Blastp searches with an E-value cutoff of 1 × 10^−5^. The retrieved-P450 candidates from *P. akamusi* were further confirmed with the support of the HMMPfam domain of P450s (PF00067, downloaded from http://pfam.xfam.org/, accessed on 15 December 2021). A set of P450s from *P. akamusi* were eventually identified based on combined sequences from the above-described methods, followed by the depletion of redundant and incomplete sequences. The gene names of retrieved chironomid P450s were assigned according to the evolutionary scheme with the standardized criteria provided by the CYP Nomenclature Committee [29].

The genome annotation gff3 files were subsequently employed to acquire physiochemical information including gene ID, chromosome location, strand, gene and CDS length, exon number, as well as the length of amino acids. The theoretical isoelectric point and average molecular weight were predicted for each P450 member by submitting sequences to Expasy (https://web.expasy.org/cgi-bin/compute_pi/, accessed on 15 December 2021).

### 2.2. Sequence Alignment, Conserved Motif Visualization, and Phylogenetic Analysis

In total, P450 amino acid sequences retrieved from *P. akamusi* were firstly aligned using ClustalW in MEGA software (ver. 10.0), after which the aligned fasta file was uploaded to WebLogo (http://weblogo.threeplusone.com/create.cgi, accessed on 20 December 2021). Five typical motifs of insect P450s were chosen to detect and visualize the conservation pattern, including Heme-Loop (F-x-x-G-x-R-x-C-x-G), Helix-K (E-x-L-R), Helix-C (W-x-x-x-R), Helix-I (A/G-G-x-D/E-T-T/S), and Meander Coil (F-x-P-E-R).

The multiple sequence alignment was also performed with the incorporation of the ClustalW algorithm by taking a total of 279 P450 amino acid sequences into account, including *D. melanogaster* (n = 86), *Chironomus tentans* (*n* = 19), *Anopheles gambiae* (*n* = 110), and *P. akamusi* (*n* = 64). The phylogenetic estimation was then analyzed on the basis of the neighbor-joining method, a distance-oriented measurement, with the aid of MEGA. A bootstrapping test (1000 replicates) was used to ensure the reliability of the tree topology. The final application of the Evolview webserver aimed to display a more intelligible and visualized tree.

### 2.3. Protein Domain, Gene Structure, and Architecture of Conserved Motifs

The Pfam database was chosen as a resource to characterize domain graphics in targeted protein sequences (http://pfam.xfam.org/, accessed on 12 December 2021). The gene annotation file together with genomic sequences of *P. akamusi* provided a graphical representation of structure features within genes, such as specified coding segments, exon/intron boundaries, and positions. MEME programs facilitated the identification of conserved motifs based on the input sequences. The parameters were predefined as an optimized motif width from 5 to 50 amino acids as well as a motif number of 10.

### 2.4. Chromosome Localization, Gene Duplication, Collinearity Analysis, and Ka/Ks Calculation

The length of four chromosomes and position information of genes were obtained from the *P. akamusi* genome annotation file. MCscanX was used to analyze the gene duplication events [30]. The Circos in TBtools endowed a visualized collinearity map, in which duplicated gene pairs were displayed among retrieved P450s [31]. A toolkit named KaKs_Calculator 2.0 was used to calculate nonsynonymous (Ka) and synonymous (Ks) substitution rates for the purpose of assessing selective pressures on target genes.

### 2.5. Culture and Treatment of P. akamusi Larvae

Chironomid larvae harvesting from field sites were cultured in glass aquaria filled with aerated de-chlorinated tap water with a controlled temperature ranging from 22 to 24 ℃. Rifampicin reagents (purity: 98%, CAS Number: 13292-46-1) were purchased and dissolved in distilled water with 10% alcohol as the co-solvent. A period of 24 h acclimation was required for larvae before receiving the xenobiotic challenge and the whole procedure took place without food supplementation. A dose of 1 mg/mL rifampicin was selected to dramatically reduce bacterial colonizers in chironomid larvae gut tissues and no harmful impacts were caused on individuals during the whole procedure. A number of *P. akamusi* larvae were transferred to a beaker and immersed in 1 mg/mL rifampicin solvent for 48 h, after which the subjects were considered as gut microbiota-deficient larvae (GD) based on the quantification data of bacterial load. Samples incubated in sterile de-chlorinated tap water with 10% alcohol were synchronously set as the gut microbiota conventional group (CV). A stock solution of CPF (100 μg/L) was prepared in DMSO (no less than 0.1%) with a gradient dilution manner. Under an aerated circumstance, the CV and GD larvae (*n* = 30) were axenically maintained and exposed to two working concentrations of CPF (5 and 50 μg/L) for 96 h. The chosen dosage was set with reference to the acute toxicity result (Supplementary Table S1). CPF was replaced by 0.1% DMSO to concurrently treat CV and GD larvae for 96 h as the control. Three biological replicates were included in each group to maximize statistical power. During the 96 h treatment of CPF, larvae demonstrating good vital signs were regarded as alive and collected for tissue dissection afterwards. Survival curves were established with the Kaplan–Meier method (log-rank test). In summary, 6 groups were set for subsequent analysis, including CV with 0.1% DMSO (CVC), GD with 0.1% DMSO (GDC), CV with 5 μg/L CPF (CV-5 μg/L CPF), GD with 5 μg/L (GD-5 μg/L CPF), CV with 50 μg/L CPF (CV-50 μg/L CPF), and GD with 50 μg/L CPF (GD-50 μg/L CPF).

### 2.6. Quantification of Gut Bacteria in P. akamusi Larvae

Larvae after rifampicin treatment were anesthetized on ice for 3–5 min. The surface of each larva was thoroughly wiped with 75% ethanol followed by 0.25% NaClO for 1 min and three times of rinsing, resulting in a complete elimination of adhering contaminants. A pool of 5 guts was firstly homogenized in 100 μL sterile PBS (pH 7.4) after the aseptic dissection procedure. A 1:1000 serial dilution was performed and 100 μL of diluted homogenates was then spread on LB agar plates. For cultivable bacteria of chironomid guts, a short incubation of 3 days at 30 ℃ was required prior to the colony forming unit (CFU) counting assay. The Ezup Column Bacteria Genomic DNA Purification Kit (Sangon Biotech, Shanghai, China) was applied to extract genomic DNA from gut tissues of *P. akamusi* larvae and the experimental steps were in line with manufacturer’s protocol. The bacterial load for each group was estimated by amplifying the fragment of 16S ribosomal RNA (16S rRNA) and calculating precise copy numbers with the conduction of qPCR. AceQ qPCR SYBR Green Master Mix (Vazyme, Nanjing, China) and the universal bacteria primers (331F and 797R, Table 1) were used in the system. Significant differences between multiple independent groups were evaluated via Student’s *t*-Test.

### 2.7. RNA Extraction from Gut Tissues and Transcriptome Analysis

Ten larval guts removed from each group were quickly ground into powders and RNA extraction from pooled tissues was then conducted with TRIzol reagent. Non-denaturing agarose gel coupled with the Agilent 2100 Bioanalyzer allowed the final measurement of qualified RNA. The Illumina ® sequencing platform at Biocloud (Beijing, China) facilitated an integrated RNA-Seq workflow. Briefly, the mRNA was isolated and enriched from total RNA using oligo (dT) attached to magnetic beads. A fragmentation buffer was applied to randomly interrupt the mRNA into shorter pieces and the fragmented mRNA was primed with random hexamers and then converted to the first strand of cDNA. AMPure XP beads were mixed with the second strand synthesis reaction for cDNA purification. Steps including end repair, polyA-tailing, adaptor ligation, and size screening (preferentially 240 bp in length) were completed in order and cDNA was eventually constructed using the PCR-based method.

Raw data were processed to obtain clean reads by excluding adapter contamination as well as low-quality nucleotides, after which compatible reads were then aligned to the *P. akamusi* reference genome (Bioproject accession: PRJNA698390) using HISAT2. We finally acquired an efficiency mapping covering more than 88% of the genomic assembly. The DESeq2 R package provided a statistical routine to indicate whether the transcripts of certain genes were differentially accumulated. The false discovery rate (FDR) < 0.01 and fold change ≥2 were adopted as the thresholds for defining the differentially expressed genes (DEGs).

### 2.8. RT-qPCR Validation for Candidate Differentially Expressed Genes

A 20 μL reaction system was prepared for each sample with triplicate assays, which contained 10 μL SYBR Green Master mix (Takara, Shanghai, China), 0.4 μL former primer (10 μM), 0.4 μL reverse primer (10 μM), 3 μL cDNA template, as well as 6.2 μL ddH2O. A handful of primers for targeted genes are listed in Table 1. The relative gene expression levels were calculated by normalizing the Ct value of each sample to that of housekeeping β-actin gene with the aid of the 2^−ΔΔCt^ method [32].

## 3. Results

### 3.1. Depiction of the P450 Multigene Family in P. akamusi

**Figure 1:**
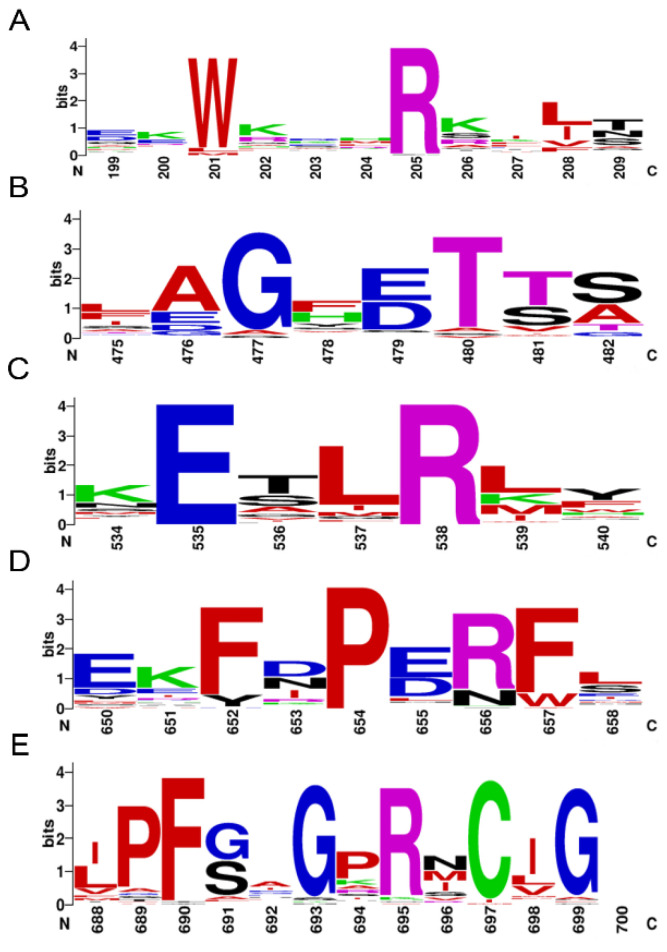
Motif pattern among PaP450s. The X-axis represents the position of amino acids and the highest letter shown in the image refers to the relative frequency of the particular amino acid at a given position. (**A**) Helix-C (W-x-x-x-R), (**B**) Helix-I (A/G-G-x-D/E-T-T/S), (**C**) Helix-K (E-x-L-R), (**D**) Meander Coil (F-x-P-E-R), and (**E**) Heme-Loop (F-x-x-G-x-R-x-C-x-G).

A total of 64 P450s were determined from the genome sequences of *P. akamusi*, abbreviated as PaP450s and named according to the standard nomenclature (Supplementary Table S2). All PaP450s were grouped into 25 families and 53 subfamilies with reference to the sequence similarity. The bioinformatics results showed that the predicted gene length of retrieved P450s ranged from 1109 to 8032 bp. Moreover, the predicted length of targeted amino acids ranged from 346 (PaCYP6AAA1P) to 623 (PaCYP6ZY1) with an average value of 499 whereas the average molecular weight was approximately 57.8 KDa, the calculation of which was based on 65 values ranging from 40.3 to 72.4 KDa. Other chemiphysiological characteristics, including chromosome location, strand, gene, and CDS length, as well as the predicted theoretical isoelectric point were also assessed and specifically enumerated in Supplementary Table S2. Five conserved motifs were depicted using multiple sequence alignment (Figure 1), which were Heme-Loop (F-x-x-G-x-R-x-C-x-G), Helix-K (E-x-L-R), Helix-C (W-x-x-x-R), Helix-I (A/G-G-x-D/E-T-T/S), and Meander Coil (F-x-P-E-R).

### 3.2. Phylogenetic Distribution and Group Clustering of Putative P450 Genes

**Figure 2:**
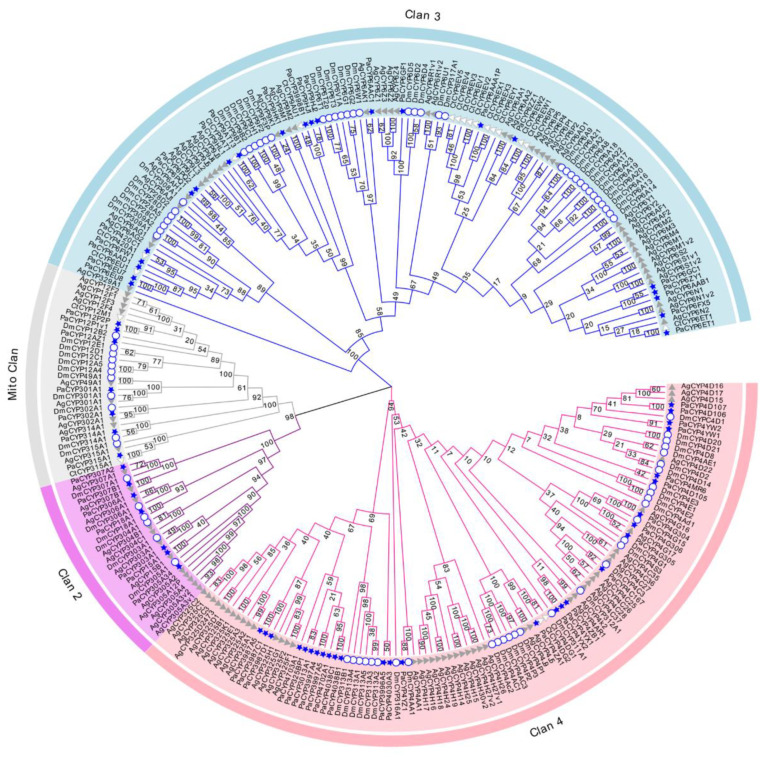
Neighbor-joining tree constructed on the basis of P450s derived from *P. akamusi* (Pa) and a set of confirmed P450s in *D. melanogaster* (Dm), *C. tentans* (Ct), and *A. gambiae* (Ag). Bootstrap values with 1000 replicates are marked on the branches. Different colors stand for four assigned clans, namely Clan 1, Clan 2, Clan 3, and Mito Clan. P450 genes marked with blue stars, white circles, white triangles, and grey triangles represent those annotated from *P. akamusi*, *D. melanogaster*, *C. tentans,* and *A. gambiae*, respectively.

The evolutionary investigation of P450s derived from *P. akamusi* was conducted with a set of confirmed P450s in *D. melanogaster*, *C. tentans*, and *A. gambiae* (Figure 2). Specifically, 279 P450 members screened from four Diptera species were aligned together and comprehensively analyzed to pinpoint the evolutionary relationship coupled with the divergent trend of P450 families in *P. akamusi* based on the neighbor-joining method. In total, 64 full-length PaP450s were allocated into four multi-family clans (Clan 2, Clan 3, Clan 4, and Mito Clan), and they were strongly supported with bootstrap values above 94%. From this clan-level classification, an overwhelming majority of the chosen genes were positioned in Clan 3 and Clan 4 with subdivision of 14 families and 40 subfamilies. In particular, 22 out of 64 PaP450s could be designated as genes affiliated to Clan 3 and further organized into 4 families and 19 subfamilies. Meanwhile, the abundant Clan 4 was composed of 28 PaP450 (42%) sequences, consisting of 10 families and 21 subfamilies. A rapid expansion of PaP450 genes in Clan 3 and Clan 4 was apparent and functional roles of these genes could be of great importance. There could be a paralogous origin between Clan 2 and Mito Clan since they appeared to generate from a mutual ancestor. However, Clan 2 and Mito Clan accounted for only less than a quarter of the total genes. No P450s from *C. tentans* were included in Clan 2, indicating the loss of certain families during the generative history of *C. tentans*. It is also worth noting that PaCYP6ET1, PaCYP9AT13, PaCYP302A1, PaCYP315A1, PaCYP303A1, PaCYP307B1, PaCYP306A1, PaCYP18A1, and PaCYP15A1 were proposed to have strict 1:1 orthologues in other surveyed counterparts.

### 3.3. Description of Gene Structures and Conserved Motif Patterns Analyzed from PaP450s

**Figure 3:**
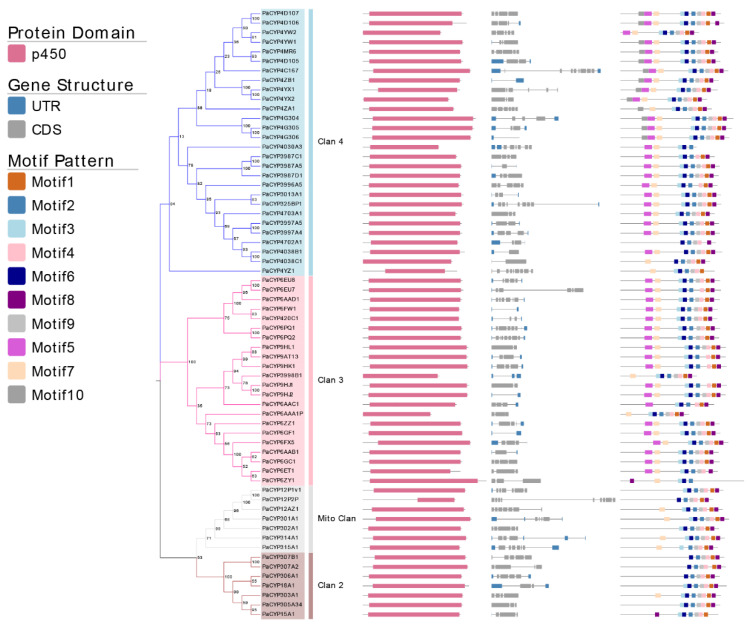
Analysis of 64 PaP450 genes, including the phylogenetic tree, domain, gene structure, and motif visualization. Different shades of the same color (blue, pink, grey, and brown) represent four distinct clans.

The loss and the gain of introns could be one of pivotal indicators to understand the evolution of gene family. The intron number of identified PaP450s ranged from minimum 0 to maximum 9 (Figure 3). Only PaCYP4G306 belonging to Clan 3 was intron-less whereas 19 out of 65 putative genes presented an intron number equal to or more than 5. Eight PaP450s had only 1 intron and all of them belonged to either Clan 3 or Clan 4, manifesting a moderately conserved scheme. All PaP450 genes belonging to Mito Clan contained a relatively large number of both introns (4–8) and exons (5–9). Taken together, a highly conserved intron/exon arrangement was observed among PaP450 genes falling in the same clan.

Moreover, the majority of PaP450s belonging to the same subgroup generally presented a great consistency of domain and motif compositions (Figure 3), which was strong adduced evidence to support the phylogenetic relatedness of P450s derived from *P. akamusi*. The Pfam analysis showed that all genes contained the p450 domain. Meanwhile, a relatively obvious discrepancy of motif pattern was observed among genes that fell into distinct clans. For example, the clade-dependent motif 10 was exclusively exhibited in the CYP4 family of Clan 3 whereas 11 out of 14 PaP450s belonging to the CYP4 family contained all 10 motifs. There were only 5 motifs residing in the PaCYP12P2P gene and its coding regions were partitioned with a long intronic insertion, thus, ending up with a shorter and less complete p450 domain. The lack of motif 5 in Clan 2 and Mito Clan together with an exclusion of motif 7 in Clan 2 could be seen as appreciable differences when a comprehensive comparison was made among all achieved genes in four clans (Figure 3). The structural variations within different families and subfamilies could imply diverse functions of genes. In other words, members falling into same subfamily, to some extent, might be endowed with similar functions.

### 3.4. Analysis of Gene Duplication and Chromosomal Localization

**Figure 4:**
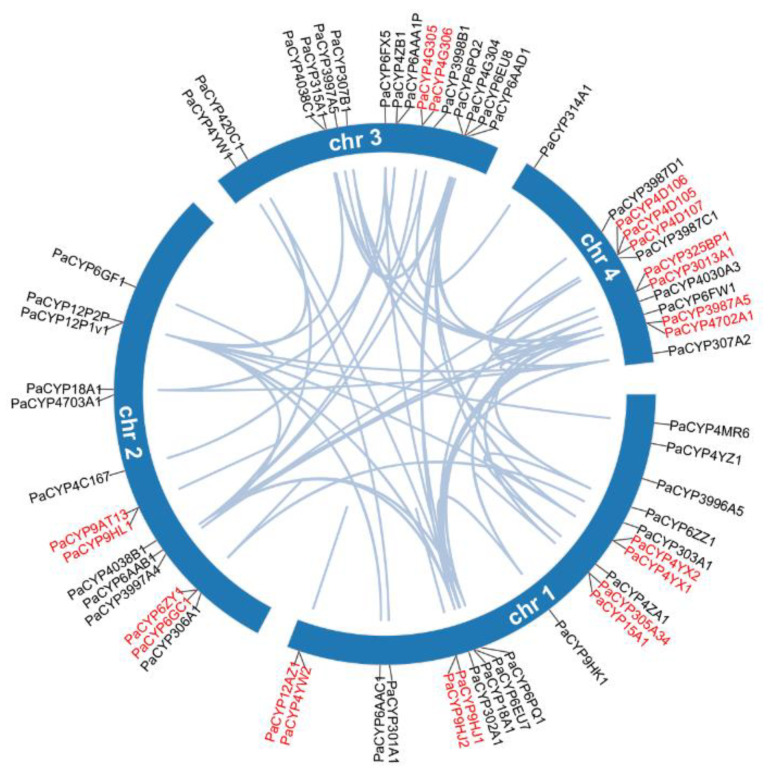
Collinearity analysis of the PaP450 family genes and their specific locations based on chromosome-level investigation. Genes marked in red demonstrate the tandem duplicated pairs in a single chromosome.

All 64 PaP450s were mapped across four chromosomes and the visualized result illustrated that chromosome 1 hosted the most PaP450 genes (21, 32.8%) while the other three chromosomes harbored 14, 16, and 13 corresponding genes, respectively (Figure 4). This chromosomal localization presented a heterogeneous scattering pattern of gene distribution over four chromosomes across the whole genome of *P. akamusi*.

Segmental and tandem duplication are necessary events that possibly prompt the augmentation of gene families. The co-linear analysis showed that 21 genes were tandemly organized into 10 groups, among which 4, 2, 1, and 3 clusters of paired genes were located on chromosomes 1, 2, 3, and 4, respectively (Figure 4). In addition, 54 segmental repeat sequences were identified as well. The Ka/Ks value was computed to clarify selective evolutionary pressure of all duplicated gene pairs and the ratio for each gene pair was < 1, which was an indicative sign for negative purifying selection during the slowly evolving course (Supplementary Table S3).

### 3.5. Establishment of Gut Microbiota-Deficient Larvae

**Figure 5:**
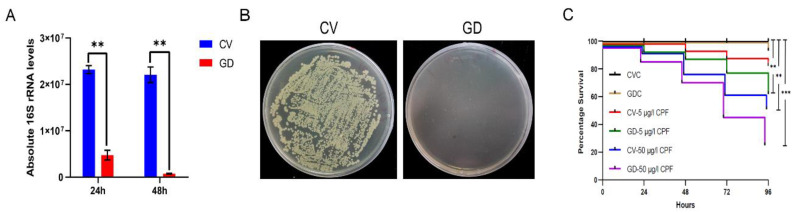
Establishment of gut microbiota-deficient larvae. The efficacy of eliminated microbial colonizers determined by (**A**) examining the absolute expression level of 16S rRNA genes from chironomid larval gut homogenates (*n* = 5) with the support of universal primers and by (**B**) culturing gut homogenates (*n* = 5) on LB agar plates. (**C**) The altered susceptibility of larval subjects to distinct conditions. CV, conventional gut community chironomid larvae; GD, gut microbiota-deficient chironomid larvae. CVC refers to CV samples treated with 0.1% DMSO while GDC stands for GD samples treated with 0.1% DMSO. The number followed by the dash in each group represents the working concentration of invading CPF. **, *p* < 0.01; *** *p* < 0.001.

We treated the 4^th^ *P. akamusi* larvae with 1 mg/mL rifampicin for 48 h to obtain individuals with deficient gut microbiota and aimed to discover the impacts of imbalanced bacteria on detoxification ability. The efficacy of establishing the gut microbiota-deficient model was examined using qPCR and the conventional culture method. The absolute quantification result showed a significant reduction in 16S rRNA copies in GD larvae 24 h and 48 h post-treatment while the elimination of cultivable microbes was confirmed with a CFU assay (Figure 5A,B).

### 3.6. The Disruption of Gut Bacterial Communities Influenced the Survivorship of P. akamusi

We examined whether CV and GD larvae reacted differently to invading CPF (Figure 5C). The survivorship of GDC larvae during a period of 96 h was consistent with that of CVC ones, suggesting that treatment with antibiotics was not able to cause negative effects on the chironomid lifespan. Moreover, the casualty did not significantly vary between CVC subjects and those incubated in the 5 μg/L CPF solution, implying a sublethal dosage of toxin. However, a concentration of 50 μg/L introduced conspicuously increased mortality in CPF-exposed normal larvae (*p* < 0.01, log-rank test). In response to either 5 or 50 μg/L CPF, the GD subjects displayed a more severe mortality rate when compared to their counterparts without the absence of gut microbiota (*p* < 0.001, log-rank test).

### 3.7. The Expression Profile of PaP450s in Gut Tissues Determined by RNA-Seq and RT-qPCR

**Figure 6:**
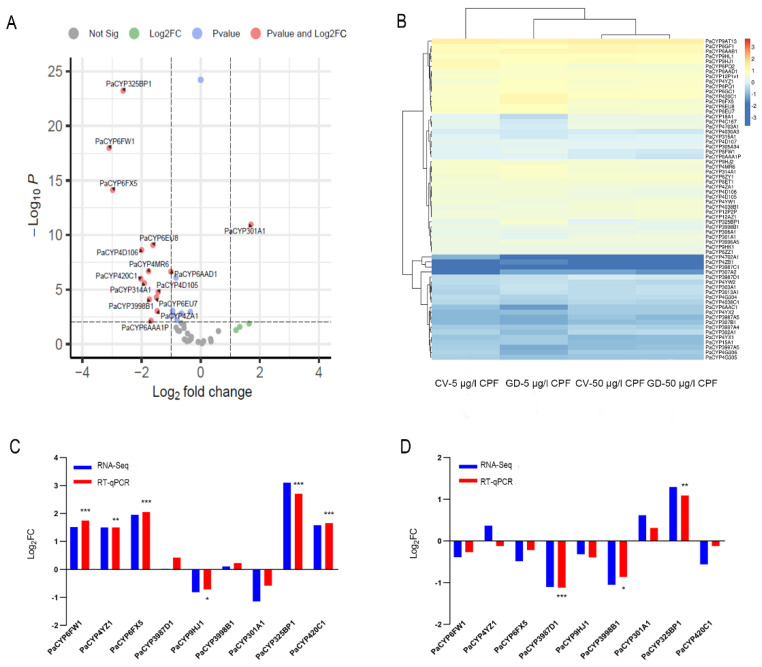
Differential expression pattern of PaP450 genes in digestive tracts of *P. akamusi.* (**A**) The altered expression of PaP450 genes in GD larvae treated with 50 μg/L CPF when compared to those treated with 5 μg/L CPF based on RNA-Seq results. Log_2_ (fold changes of FPKM values) is indicated on the X-axis; the negative Log_10_ (*p*-value) of the t-test for the fold change between two groups is on the Y-axis. (**B**) Heatmap revealing the expression pattern based on the transcriptome data of four groups. (**C**) RNA-Seq and qPCR data revealing the expression pattern of 9 PaP450s between GD larvae treated with 5 μg/L CPF and CV larvae treated with 5 μg/L CPF. (**D**) RNA-Seq and qPCR data revealing the expression pattern of 9 PaP450s between GD larvae treated with 50 μg/L CPF and CV larvae treated with 50 μg/L CPF. The absolute Log_2_FC value of RNA-Seq greater than 1 is considered as significant. Asterisks denote statistically significant differences in the RT-qPCR (t-test; * *p* < 0.05; ** *p* < 0.01; *** *p* < 0.001).

We then investigated whether PaP450s differed between CV and GD samples subjected to CPF. It was revealed that no rifampicin-responsive PaP450s in gut tissues dissected from GDC and GNC larvae were significantly different at 96 h, presenting an indistinctive influence of antibiotics on P450 genes (Supplementary Table S4). The transcriptome data showed that none of the identified 64 PaP450s displayed remarkable discrepancy when the dosage of challenged CPF elevated from sublethal to lethal, indicating that the expression profiles of PaP450s in digestive tracts were not associated with an augmenting concentration of xenobiotics (Supplementary Table S4). However, 14 retrieved PaP450s (PaCYP325BP1, PaCYP4D106, PaCYP6AAA1P, PaCYP6FW1, PaCYP3998B1, PaCYP4MR6, PaCYP6EU7, PaCYP6FX5, PaCYP6AAD1, PaCYP4D105, PaCYP314A1, PaCYP4ZA1, PaCYP6EU8, and PaCYP420C1) in GD larvae guts were noticeably attenuated while PaCYP301A1 was solely upregulated when the concentration of challenging CPF was augmented from 5 to 50 μg/l (log2FC < −1, *p* value < 0.01) (Figure 6A, Supplementary Table S4).

Certain P450s presented expression changes between CV and GD larvae challenged with CPF in a dosage-dependent manner (Figure 6B). Among the above-mentioned 14 differentially expressed genes, 6 PaP450s were discovered with a significant alteration when a comparison was invited between CV and GD samples responding to 5 μg/L CPF. According to the transcriptome data, a concentration of sublethal CPF resulted in an obvious suppression of PaCYP301A1 coupled with an upregulation of PaCYP6FW1, PaCYP4YZ1, PaCYP6FX5, PaCYP325BP1, and PaCYP420C1 in GD larval guts when compared with CV ones (|log2FC| > 1, *p* value < 0.01). RT-qPCR of targets provided consistent data with the exception of PaCYP301A1 (Figure 6C, Supplementary Table S5). Meanwhile, 50 μg/L CPF induced a remarkably lower expression of PaCYP3987D1 and PaCYP3998B1 in digestive tracts of GD larvae than CV ones, which agreed with the RT-qPCR data (Figure 6D, Supplementary Table S5). To note, both sublethal (5 μg/L) and lethal (50 μg/L) CPF generated a significantly higher level of PaCYP325BP1 in the GD group than the normal one with a fold change of approximately 8- and 2.5-fold, respectively. Collectively, an elevated tread was observed in GD subjects incubated in sublethal CPF while a decreasing tendency was more common in those treated with a lethal dosage, implying that the expression of gut microbiota-regulated PaP450s in guts was dependent on toxin level and presented distinct patterns.

## 4. Discussion

### 4.1. Comprehensive Identification and Analysis of P450s in P. akamusi

Insect P450 enzymes are responsible for catalyzing physiological reactions, from metabolism of foreign chemicals to the biosynthesis of ecdysteroids and juvenile hormones. The ascribed detoxified functions of this enzyme superfamily have been receiving considerable attention and numerous studies have notably reported that P450-associated clearance or conversion of toxic compounds could confer resistance or adaptation to xenobiotics in insects [33]. Considering that characterization of P450s in specific insects is the primary step towards unravelling P450-involved responses to stressful stimuli, we first conducted a genome-wide identification and analysis of P450s in chironomid *P. akamusi*.

A total of 64 P450s were identified from the release of the whole genomic sequence and were denominated with unique names in terms of a unified nomenclature principle [29]. All the retrieved sequences were classified into four well-supported clans and 25 families (subcomponents of clans) based on the sequence identity with published members (Supplementary Table S2). A comparative survey exerts that within insects, some species carry a much larger repertoire of P450s than that of *P. akamusi*, such as *C. quinquefasciatus* (196), *Aedes albopictus* (186), and *Nicrophorus vespilloides* (129), while *Aculops lycopersici*, *Calopteryx splendens*, *P. humanus,* and *Apis mellifera* possess a relatively small CYPome size of no more than 50 [34]. The varied size of P450s might result from an evolutionary differentiation of diverse species or the ecological niche in which insects survive and evolve. An apparent expansion of PaP450s in Clan 3 (34%) and Clan 4 (28%) is a characteristic feature in *P. akamusi* (Figure 2), which is in accordance with the allocation of P450s across four clans in other insects. In particular, emerging evidence has proved that the recruitment of P450s in Clan 3 and Clan 4 could be deemed as an adaptive tactic that insects have developed to cope with exogenous xenobiotics. For instance, virtually all P450 members of Clan 3 that were discovered in *P. xylostella* and *H. armigera* could act as insecticide metabolizers and function in the oxidative transformation of deleterious xenobiotics [35]. Therefore, we speculate that a relatively large number of *P. akamusi* P450s (62%) within Clan 3 and Clan 4 are indicative of protective strategies against deleterious compounds.

Sub-genomic duplication events, such as segmental and tandem duplications, are suggested as the main driving force of gene family expansion and to some extent, could prevent function loss provoked by genetic mutations [36]. The chromosome-level investigation of the P450 superfamily in *P. akamusi* was conducted and the result showed that 21 of 64 (33%) were tandemly positioned, with chromosome 1 housing the highest density of homologous pairs. Additionally, sets of 4, 2, and 7 repeats were clustered into tandem arrays residing in chromosomes 2, 3, and 4, respectively (Figure 3). Interestingly, a CYP12 subfamily member (PaCYP12AZ1) belonging to the Mito Clan and a member (CYP4YW2) from the Clan 4 lay adjacent to each other and were tandemly positioned on chromosome 1. However, this is ambivalent over a consensus that tandemly duplicated gene pairs commonly end up belonging to the same subfamily, or at least the same clan. Previous research has suggested that tandem duplicated genes are enriched in resistance pathways and highly related to stress responsiveness [37]. We hypothesize that the presence of a moderate-scale tandem duplication in *P. akamusi* could be a necessary source for genes to acquire defense-responsive functions, which might aid midges’ acclimation to pollutant-rich environments.

### 4.2. The Possible Interaction between Bacterial Colonizers and P450s in P. akamusi Larval Guts

CPF, an organophosphate insecticide, could cause detrimental effects on brain activities by irreversibly binding to acetylcholinesterase (AChE) and is globally applied for agricultural and residential deinsectization. However, exclusive of the beneficial effects from CPF on intended targets, other aquatic life could be disproportionately influenced by CPF, especially when this neurotoxicant agent flows into water systems (surface water, rivers, groundwater, etc.) and remaining at concentrations exceeding the allowable levels [38]. Some aquatic creatures are quite sensitive to CPF (marine mysid has 96 h-LC_50_ of 0.026 μg/L) [39] while freshwater mussels are able to escape from the toxic influences generated by CPF with its 96 h-LC_50_ reaching up to 50,000 μg/L [40]. The chemical toxicity of CPF is also highly variable among insects. For example, the 48 h-LC_50_ of chlorpyrifos against blackfly and the chironomid *Diamesa zernyi* is 0.28 and 5.24 μg/L, respectively. In this study, the medium lethal concentration of CPF was determined on the 4^th^ *P. akamusi* larvae and the 96 h-LC_50_ value was reported as 68 μg/L (Supplementary Table S1). A largely discrepant range for LC_50_ values reveals the truth that chemical toxicity conspicuously differed among different species and the underlying mechanism used by each species to cope with this toxic agent is unique, but most of them still remain ambiguous.

The insect gut is a primary site for the body to detoxify unexpected pesticides [41]. There is a dynamic cross-talk between the microbial flora residing in digestive tracts and hosts to provide the body with a reservoir of functions, such as nutrition acquisition, immune responses, detoxification, etc. [42]. The measurement of bacterial copies coupled with conventional cultivation demonstrated that 48 h of 1 mg/mL rifampicin treatment was able to significantly diminish the microbial community in *P. akamusi* larvae and thus gut microbiota-deficient (GD) individuals were successfully established (Figure 5). The mortality of both GD and CV larvae were evaluated during 96 h CPF treatment and the result depicted that, irrespective of the concentration, larvae with normal gut microbiota were more capable of surviving than samples with reduced bacterial colonizers. Our data revealed that the antibiotic treatment could accelerate the mortality of CPF-challenged larvae (Figure 5), similar to previous studies [25]. In short, 1 mg/mL rifampicin was suitable to impair the bacterial colonizers without causing unnecessary loss and gut microorganisms could play a part in ameliorating insecticide-induced damage.

Studies regarding the interplay between gut microbiota and host detoxification ability are currently a point of interest. It has been recognized that intestinal microflora is considered as a mediator for indirect xenobiotic metabolism. Here, we attempted to illustrate how the lack of gut-associated microbial colonizers altered the expression of detoxified enzymes with a focus on well-researched P450s when larvae confront foreign substrates. Previous research has reported that 100 μg/L benzopyrene triggered a higher number of P450 genes than 10 μg/L benzopyrene did, indicating that the elevated impression of P450s could initiate an intensified metabolism of more incremental chemicals [43]. Compiled from our transcriptome data, all retrieved P450s were held constant in normal gut tissues with an increased prevalence of CPF from 5 to 50 μg/L, while the increasing pesticides induced a remarkable downregulation of 14 P450 isozymes in larval guts devoid of microbial colonizers (Figure 6). Meanwhile, depletion of bacterial symbionts gave rise to a sharper death rate when larvae experienced a 10-fold increasing concentration of CPF. Previous investigations have also revealed that detoxified enzymes in insect gut tissues could mitigate the toxic effects of pesticides. For instance, the midgut-specific expression of CYP340s in diamondback moth was proved to be connected to abamectin resistance [44], and a prominent role of CYP6G1 expressed in *D. melanogaster* midguts was also highlighted in pesticide toxicology [45]. Combined with our finding, we preliminarily assume that the imbalanced microbiota introduced an adverse influence on larval survivorship and this consequence might be associated with the downregulation of 14 P450 genes in guts (PaCYP325BP1, PaCYP4D106, PaCYP6AAA1P, PaCYP6FW1, PaCYP3998B1, PaCYP4MR6, PaCYP6EU7, PaCYP6FX5, PaCYP6AAD1, PaCYP4D105, PaCYP314A1, PaCYP4ZA1, PaCYP6EU8, and PaCYP420C1).

Since no difference was discovered between GD and CV larvae, we considered a negligible role of antibiotics on the expression level of P450s (Supplementary Table S3). Of these 14 P450s, it was noteworthy that the sublethal dosage of CPF leads to significantly increased expression, to varying degrees, of PaCYP6FW1, PaCYP6FX5, PaCYP325BP1, and PaCYP420C1 in GD larvae (Supplementary Table S3, Figure 6). This is in accordance with a similar study on mice that revealed a polybrominated diphenyl ethers (PBDEs)-mediated upregulation of several CYP proteins in the colon and liver of germ-free subjects [46]. There is a set of detoxifying pathways that stem from the microbial communities of *C. ramosus* when individuals are exposed to environmental pollutants, such as heavy metals or harmful xenobiotic, suggesting a positive role of endogenous microbiota in protecting chironomids from stress situations [20]. Certain bacteria strains isolated from insect guts are capable of bioremediation against target compounds and the lack of gut symbionts could increase xenobiotic metabolites [47]. For example, *Flavobacterium* can facilitate the degradation of CPF [48]; this strain happened to be a core member of gut microbiota in *P. akamusi* and remarkably proliferated with invading CPF (unpublished data). We, therefore, suppose that the lack of most bacterial colonizers might passively affect CPF metabolism and the microbiome-derived metabolites possibly have localized effects, such as stimulating certain P450s. Another possible conjecture is that the elevated P450s might be triggered by gut dysbiosis and acted as a stress-responsive modulation to defend against invading xenobiotics during a short period of time, reflected by the lethal assay data showing that the 5 μg/L pesticide slightly elevated the mortality rate of GD larvae compared to that of CV ones. Undoubtedly, continuous studies are warranted to elucidate the precise mechanism and further investigations regarding the metabolism managed by certain gut microbes and candidate PaP450s should be implemented.

The co-occurrence of gut dysbiosis and lethal dosage of CPF treatment resulted in high mortality of *P. akamusi* larvae (*ca.* 90%) and an obvious downregulation of PaCYP3998B1 and PaCYP3987D1 (Figure 6). One previous report has proved that antibiotic-triggered gut dysbiosis could give rise to attenuated expression of P450s in the midguts of insecticide-exposed honey bees as well as a higher casualty rate [25], which strongly agrees with our data. The decreased level of PaCYP3998B1 and PaCYP3987D1, to some extent, reflected impaired host endogenous detoxification capability potentiated by gut dysbiosis. Thus, gut microbiota of chironomid larvae could be considered as a modifier for CPF-mediated regulation of P450s in digestive tracts and the altered pattern is relevant to the concentration of invading CPF.

## 5. Conclusions

The present study is the first to isolate and characterize a total of 64 P450 genes in chironomid species based on a comprehensive database survey. Gene structure, phylogenetic evolution, and conserved motif pattern were thoroughly examined. Insufficient gut microbiota in CPF-treated *P. akamusi* larvae introduced a significantly varied pattern of certain PaP450s in a dose-dependent manner compared to the normal individuals, suggesting a potential role of gut microbial commensals in regulating local P450s. The severe mortality of gut microbiota-deficient larvae driven by a lethal concentration of CPF might be associated with the remarkable downregulation of PaCYP3998B1 and PaCYP3987D1. This study could lay a preliminary foundation for us to further explore the combinatory and synergistic effects of gut symbionts and detoxified enzymes on pollution-tolerant chironomid species.

## Figures and Tables

**Table 1:** Oligonucleotide primers used for the amplification of targeted genes in the qPCR assay.

Primer Name	Sequence (5′–3′)
16S rRNA-331F	TCCTACGGGAGGCAGCAGT
16S rRNA-797R	GGACTACCAGGGTATCTAATCCTGTT
Paβ-actin-F	TCTTCCAGCCATCCTTCTTG
Paβ-actin-R	CGGTGATTTCCTTCTGCATT
PaCYP6FW1-F	TCCAGACACCTACCGCCAACTC
PaCYP6FW1-R	AACCGTCCTCGACCACTCTGTAG
PaCYP4YZ1-F	TGTGTCAACTCTGCCTGTGCTTAC
PaCYP4YZ1-R	TCGCCTCATACCTCTGGAACGG
PaCYP6FX5-F	ACGAAGAGCGGTGATGACAAGTG
PaCYP6FX5-R	AACTGTCCGAAGCAGGCGAATATC
PaCYP3987D1-F	GCTAATGCTGCCCAGGTCTCAAC
PaCYP3987D1-R	CTCGCTCAACTTCCACAACCTATCC
PaCYP9HJ1-F	CCATCGACCCAAACCTGAAGACTG
PaCYP9HJ1-R	TAGGCAGACGGCTTGAGGCTAG
PaCYP3998B1-F	ACGCCTTCTCTACGCCTTCTCC
PaCYP3998B1-R	GGTAGGTAGGTGGTCGGTCGTC
PaCYP301A1-F	ACAAGAAGGGCGTGCGTCAAAC
PaCYP301A1-R	GCAGCAGACAAGCCAGGTTGAG
PaCYP325BP1-F	ACCACATCAACATCATCACCACCTG
PaCYP325BP1-R	AGGAGTAGTTTGAACCAGCGGATTG
PaCYP420C1-F	GCTCCCAGGTTGCGTCTTGTTC
PaCYP420C1-R	GGCGATGGCATCTGCGTCTATC

## Data Availability

The data presented in this study are available in article and supplementary materials. The *P. akamusi* genome sequence and annotation files are available at NCBI (Bioproject accession: PRJNA698390).

## Supplementary Materials

The following supporting information can be downloaded at: https://www.mdpi.com/article/10.3390/insects13090765/s1, Table S1: The median lethal concentration (LC50) values of CPF for *P. akamusi* larvae at different time points (*n* = 30); Table S2: Detailed information with regard to P450 genes retrieved from *P. akamusi*; Table S3: Ka/Ks analysis for the duplicated PaP450 gene pairs; Table S4: The significant change in gut expressed PaP450 genes; Table S5: The comparison of gut expressed PaP450 genes between CV and GD samples.
