# Supplementary material for: Genome-Wide Identification of P450 Genes in Chironomid Propsilocerus akamusi Reveals Candidate Genes Involved in Gut Microbiota-Mediated Detoxification of Chlorpyrifos
Source: Insects. 2022 Aug 24;13(9):765. doi: 10.3390/insects13090765 (PMC9504602; doi:10.3390/insects13090765)
Supplement: Supplementary file 1 [file insects-13-00765-s001.zip › insects-1860644-supplementary.pdf]

Table S1. The median lethal concentration ($LC_{50}$) values of CPF for *P. akamusi* larvae at different time points (n=30)

| Exposure time (h) | Toxicity regression equation | Lethal concentration 50% (LC50, μg/l) | 95% confidence interval (μg/l) | Correlation coefficient |
|---|---|---|---|---|
| 24 | y=0.431x-1.638 | 6304.862 | [2.874,11.222] | 0.644 |
| 48 | y=0.497x-1.296 | 403.59 | [2.187,3.938] | 0.807 |
| 72 | y=0.801x-01.175 | 29.334 | [1.170,1.686] | 0.666 |
| 96 | y=0.977x-1.133 | 14.425 | [0.846,1.368] | 0.660 |

Table S2.Detailed information with regard to P450 genes retrieved from *P. akamusi.*

| **Clan** | **Gene ID** | **Name** | **Chromosome** | **Strand** | **Genelen** | **ExonNum** | **CDSLen** | **AALen** | **PI** | **MW (Da)** |
|---|---|---|---|---|---|---|---|---|---|---|
| Clan 2 | EVM0003603 | PaCYP15A1 | Chromosome 1 | - | 1705 | 4 | 1479 | 493 | 8.77 | 56707.38 |
| | EVM0009779 | PaCYP18A1 | Chromosome 2 | - | 3713 | 7 | 1605 | 535 | 6.72 | 61774.78 |
| | EVM0007945 | PaCYP303A1 | Chromosome 1 | - | 1746 | 7 | 1518 | 506 | 7.14 | 58558.45 |
| | EVM0001723 | PaCYP305A34 | Chromosome 1 | - | 1623 | 3 | 1509 | 503 | 8.86 | 58139.54 |
| | EVM0008860 | PaCYP306A1 | Chromosome 2 | - | 2554 | 4 | 1500 | 500 | 6.54 | 57222.54 |
| | EVM0004208 | PaCYP307A2 | Chromosome 4 | - | 3265 | 9 | 1593 | 531 | 7.13 | 60963.59 |
| | EVM0000618 | PaCYP307B1 | Chromosome 3 | - | 2602 | 3 | 1569 | 523 | 9.02 | 60184.63 |
| Clan 3 | EVM0005788 | PaCYP3998B1 | Chromosome 3 | - | 1904 | 3 | 1158 | 386 | 8.33 | 44280.39 |
| | EVM0007979 | PaCYP420C1 | Chromosome 3 | - | 1968 | 9 | 1464 | 488 | 6.62 | 56706.49 |
| | EVM0002438 | PaCYP6AAA1P | Chromosome 3 | - | 1109 | 1 | 1038 | 346 | 8.06 | 40339.01 |
| | EVM0000320 | PaCYP6AAB1 | Chromosome 2 | - | 1680 | 2 | 1485 | 495 | 8.82 | 56717.67 |
| | EVM0011176 | PaCYP6AAC1 | Chromosome 1 | - | 1738 | 4 | 1416 | 472 | 7.64 | 54195.25 |
| | EVM0005172 | PaCYP6AAD1 | Chromosome 3 | + | 2142 | 4 | 1500 | 500 | 8.34 | 58337.87 |
| | EVM0003977 | PaCYP6ET1 | Chromosome 1 | - | 1708 | 5 | 1473 | 491 | 8.32 | 56432.87 |
| | EVM0011861 | PaCYP6EU7 | Chromosome 1 | + | 5958 | 4 | 1518 | 506 | 6.46 | 59003.36 |
| | EVM0010028 | PaCYP6EU8 | Chromosome 3 | - | 1985 | 10 | 1506 | 502 | 7.21 | 58450.66 |
| | EVM0001503 | PaCYP6FW1 | Chromosome 4 | + | 1751 | 4 | 1470 | 490 | 8.15 | 56398.03 |
| | EVM0003931 | PaCYP6FX5 | Chromosome 3 | - | 2314 | 5 | 1632 | 544 | 8.45 | 63018.13 |
| | EVM0008668 | PaCYP6GC1 | Chromosome 2 | - | 1668 | 4 | 1500 | 500 | 9.18 | 57816.41 |
| | EVM0004122 | PaCYP6GF1 | Chromosome 2 | + | 1916 | 3 | 1512 | 504 | 8.78 | 57612.91 |
| | EVM0006654 | PaCYP6PQ1 | Chromosome 1 | - | 2310 | 6 | 1506 | 502 | 6.11 | 57591.64 |

| | | | | | | | | | | |
|---|---|---|---|---|---|---|---|---|---|---|
| | EVM0007561 | PaCYP6PQ2 | Chromosome 3 | + | 2178 | 4 | 1494 | 498 | 8.72 | 57067.29 |
| | EVM0009272 | PaCYP6ZY1 | Chromosome 2 | - | 3194 | 5 | 1869 | 623 | 9.00 | 72428.91 |
| | EVM0005751 | PaCYP6ZZ1 | Chromosome 1 | + | 2099 | 2 | 1500 | 500 | 8.79 | 57157.13 |
| | EVM0010649 | PaCYP9AT13 | Chromosome 2 | - | 1965 | 4 | 1590 | 530 | 6.31 | 61222.74 |
| | EVM0005770 | PaCYP9HJ1 | Chromosome 1 | - | 1698 | 4 | 1608 | 536 | 8.82 | 62234.46 |
| | EVM0007293 | PaCYP9HJ2 | Chromosome 1 | + | 1881 | 6 | 1590 | 530 | 8.48 | 61036.71 |
| | EVM0000292 | PaCYP9HK1 | Chromosome 1 | + | 2050 | 8 | 1605 | 535 | 6.32 | 62236.89 |
| | EVM0008145 | PaCYP9HL1 | Chromosome 2 | - | 1652 | 4 | 1593 | 531 | 8.15 | 61109.13 |
| Clan 4 | EVM0007469 | PaCYP3013A1 | Chromosome 4 | + | 1758 | 3 | 1521 | 507 | 6.64 | 59389.88 |
| | EVM0007575 | PaCYP325BP1 | Chromosome 4 | + | 6986 | 6 | 1524 | 508 | 8.80 | 59069.70 |
| | EVM0010725 | PaCYP3987A5 | Chromosome 4 | + | 1649 | 5 | 1500 | 500 | 8.60 | 57097.82 |
| | EVM0009536 | PaCYP3987C1 | Chromosome 4 | + | 1609 | 2 | 1425 | 475 | 8.67 | 55205.18 |
| | EVM0005113 | PaCYP3987D1 | Chromosome 4 | - | 1975 | 5 | 1485 | 495 | 7.97 | 56777.59 |
| | EVM0005667 | PaCYP3996A5 | Chromosome 1 | + | 2070 | 5 | 1467 | 489 | 8.16 | 56777.95 |
| | EVM0007735 | PaCYP3997A4 | Chromosome 2 | + | 2383 | 3 | 1500 | 500 | 6.04 | 57939.91 |
| | EVM0001371 | PaCYP3997A5 | Chromosome 3 | - | 1825 | 7 | 1491 | 497 | 8.83 | 58082.81 |
| | EVM0003672 | PaCYP4030A3 | Chromosome 4 | - | 2606 | 3 | 1152 | 384 | 6.05 | 44974.12 |
| | EVM0005123 | PaCYP4038B1 | Chromosome 2 | - | 1767 | 4 | 1539 | 513 | 8.63 | 60230.38 |
| | EVM0006012 | PaCYP4038C1 | Chromosome 3 | + | 2257 | 6 | 1356 | 452 | 8.90 | 52547.22 |
| | EVM0007072 | PaCYP4702A1 | Chromosome 4 | - | 2168 | 9 | 1443 | 481 | 8.45 | 56259.68 |
| | EVM0007161 | PaCYP4703A1 | Chromosome 2 | - | 1548 | 8 | 1425 | 475 | 8.84 | 59983.76 |
| | EVM0003657 | PaCYP4C167 | Chromosome 2 | + | 7074 | 6 | 1632 | 544 | 7.16 | 62614.29 |
| | EVM0009599 | PaCYP4D105 | Chromosome 4 | - | 2552 | 2 | 1512 | 504 | 8.00 | 58344.93 |
| | EVM0008066 | PaCYP4D106 | Chromosome 4 | - | 1889 | 4 | 1569 | 523 | 8.26 | 59534.42 |
| | EVM0005110 | PaCYP4D107 | Chromosome 4 | - | 1707 | 3 | 1518 | 506 | 6.88 | 57200.80 |
| | EVM0005550 | PaCYP4G304 | Chromosome 3 | + | 4340 | 5 | 1707 | 569 | 8.86 | 64825.04 |
| | EVM0001303 | PaCYP4G305 | Chromosome 3 | - | 2276 | 8 | 1683 | 561 | 8.95 | 64767.93 |
| | EVM0008669 | PaCYP4G306 | Chromosome 3 | - | 1816 | 5 | 1644 | 548 | 8.79 | 63005.78 |
| | EVM0001233 | PaCYP4MR6 | Chromosome 1 | + | 1785 | 4 | 1479 | 493 | 6.03 | 57149.48 |
| | EVM0010956 | PaCYP4YW1 | Chromosome 3 | + | 1717 | 5 | 1518 | 506 | 8.61 | 58614.98 |
| | EVM0007328 | PaCYP4YW2 | Chromosome 1 | + | 1464 | 3 | 1188 | 396 | 6.07 | 45462.35 |
| | EVM0006546 | PaCYP4YX1 | Chromosome 1 | - | 4313 | 2 | 1470 | 490 | 8.43 | 56293.22 |

| | | | | | | | | | | |
|---|---|---|---|---|---|---|---|---|---|---|
| | EVM0007218 | PaCYP4YX2 | Chromosome 1 | - | 1433 | 7 | 1311 | 437 | 7.13 | 50297.06 |
| | EVM0001842 | PaCYP4YZ1 | Chromosome 1 | + | 2689 | 3 | 1425 | 475 | 8.35 | 54726.39 |
| | EVM0003351 | PaCYP4ZA1 | Chromosome 1 | - | 1695 | 9 | 1380 | 460 | 5.82 | 53433.74 |
| | EVM0004611 | PaCYP4ZB1 | Chromosome 3 | + | 2089 | 4 | 1485 | 495 | 8.60 | 57002.92 |
| Mitochondrial (Mito) Clan | EVM0011060 | PaCYP12AZ1 | Chromosome 1 | - | 3272 | 4 | 1545 | 515 | 8.86 | 59491.71 |
| | EVM0000161 | PaCYP12P1v1 | Chromosome 2 | + | 2307 | 5 | 1557 | 519 | 8.84 | 59983.76 |
| | EVM0003565 | PaCYP12P2P | Chromosome 2 | + | 8032 | 4 | 1401 | 467 | 8.84 | 59878.61 |
| | EVM0005888 | PaCYP301A1 | Chromosome 1 | + | 4610 | 7 | 1641 | 547 | 8.90 | 62511.67 |
| | EVM0000338 | PaCYP302A1 | Chromosome 1 | - | 1727 | 7 | 1491 | 497 | 8.9 | 57565.30 |
| | EVM0011023 | PaCYP314A1 | Chromosome 4 | - | 6113 | 3 | 1578 | 526 | 5.96 | 60621.89 |
| | EVM0007552 | PaCYP315A1 | Chromosome 3 | - | 4371 | 4 | 1476 | 492 | 8.88 | 56607.89 |

Table S3. Ka/Ks analysis for the duplicated PaP450 gene pairs

| Duplicate pair (Gene ID) | Duplicate pair (Gene name) | Duplicate type | Ka | Ks | Ka/Ks | Purifying selection |
|---|---|---|---|---|---|---|
| EVM0000161/EVM0000338 | PaCYP12P1v1/PaCYP302A1 | Segmental duplication | 0.853 | 2.116 | 0.403 | Yes |
| EVM0000161/EVM0001233 | PaCYP12P1v1/PaCYP4MR6 | Segmental duplication | 1.015 | 2.418 | 0.420 | Yes |
| EVM0000161/EVM0001503 | PaCYP12P1v1/PaCYP6FW1 | Segmental duplication | 0.892 | 3.254 | 0.274 | Yes |
| EVM0000161/EVM0004208 | PaCYP12P1v1/PaCYP307A2 | Segmental duplication | 0.963 | 1.795 | 0.537 | Yes |
| EVM0000161/EVM0006012 | PaCYP12P1v1/PaCYP4038C1 | Segmental duplication | 0.992 | 3.764 | 0.263 | Yes |
| EVM0000161/EVM0007218 | PaCYP12P1v1/PaCYP4YX2 | Segmental duplication | 0.908 | 2.521 | 0.360 | Yes |
| EVM0000161/EVM0007735 | PaCYP12P1v1/PaCYP3997A4 | Segmental duplication | 0.904 | 4.542 | 0.199 | Yes |
| EVM0000161/EVM0007979 | PaCYP12P1v1/PaCYP420C1 | Segmental duplication | 0.887 | 1.792 | 0.495 | Yes |
| EVM0000161/EVM0011176 | PaCYP12P1v1/PaCYP6AAC1 | Segmental duplication | 0.880 | 2.135 | 0.412 | Yes |
| EVM0000292/EVM0003672 | PaCYP9HK1/PaCYP4030A3 | Segmental duplication | 0.964 | 3.861 | 0.250 | Yes |
| EVM0000292/EVM0006654 | PaCYP9HK1/PaCYP6PQ1 | Segmental duplication | 0.770 | 2.301 | 0.335 | Yes |
| EVM0000320/EVM0000338 | PaCYP6AAB1/PaCYP302A1 | Segmental duplication | 0.938 | 2.327 | 0.403 | Yes |
| EVM0000320/EVM0001503 | PaCYP6AAB1/PaCYP6FW1 | Segmental duplication | 0.749 | 2.344 | 0.319 | Yes |
| EVM0000320/EVM0004122 | PaCYP6AAB1/PaCYP6GF1 | Segmental duplication | 0.675 | 1.858 | 0.363 | Yes |

| | | | | | | |
|---|---|---|---|---|---|---|
| EVM0000320/EVM0005788 | PaCYP6AAB1/PaCYP3998B1 | Segmental duplication | 0.828 | 1.766 | 0.469 | Yes |
| EVM0000320/EVM0006654 | PaCYP6AAB1/PaCYP6PQ1 | Segmental duplication | 0.862 | 1.978 | 0.436 | Yes |
| EVM0000320/EVM0007561 | PaCYP6AAB1/PaCYP6PQ2 | Segmental duplication | 0.735 | 1.969 | 0.373 | Yes |
| EVM0000320/EVM0007979 | PaCYP6AAB1/PaCYP420C1 | Segmental duplication | 0.724 | 1.994 | 0.363 | Yes |
| EVM0000338/EVM0001503 | PaCYP302A1/PaCYP6FW1 | Segmental duplication | 1.026 | 6.004 | 0.171 | Yes |
| EVM0000338/EVM0004208 | PaCYP302A1/PaCYP307A2 | Segmental duplication | 1.009 | 2.729 | 0.370 | Yes |
| EVM0000338/EVM0005172 | PaCYP302A1/PaCYP6AAD1 | Segmental duplication | 0.970 | 1.663 | 0.583 | Yes |
| EVM0000618/EVM0004122 | PaCYP307B1/PaCYP6GF1 | Segmental duplication | 1.048 | 3.112 | 0.337 | Yes |
| EVM0000618/EVM0005751 | PaCYP307B1/PaCYP6ZZ1 | Segmental duplication | 0.965 | 2.535 | 0.381 | Yes |
| EVM0001371/EVM0007561 | PaCYP3997A5/PaCYP6PQ2 | Segmental duplication | 0.895 | 3.909 | 0.229 | Yes |
| EVM0001503/EVM0003931 | PaCYP6FW1/PaCYP6FX5 | Segmental duplication | 0.793 | 2.263 | 0.350 | Yes |
| EVM0001503/EVM0004122 | PaCYP6FW1/PaCYP6GF1 | Segmental duplication | 0.790 | 1.514 | 0.522 | Yes |
| EVM0001503/EVM0006654 | PaCYP6FW1/PaCYP6PQ1 | Segmental duplication | 0.772 | 2.188 | 0.353 | Yes |
| EVM0001503/EVM0007735 | PaCYP6FW1/PaCYP3997A4 | Segmental duplication | 0.917 | 2.690 | 0.341 | Yes |
| EVM0001723/EVM0004122 | PaCYP305A34/PaCYP6GF1 | Segmental duplication | 1.036 | 1.750 | 0.592 | Yes |
| EVM0003351/EVM0005751 | PaCYP4ZA1/PaCYP6ZZ1 | Segmental duplication | 0.850 | 2.992 | 0.284 | Yes |
| EVM0003351/EVM0010028 | PaCYP4ZA1/PaCYP6EU8 | Segmental duplication | 0.924 | 2.256 | 0.409 | Yes |
| EVM0003657/EVM0003931 | PaCYP4C167/PaCYP6FX5 | Segmental duplication | 0.812 | 1.928 | 0.421 | Yes |
| EVM0003672/EVM0007735 | PaCYP4030A3/PaCYP3997A4 | Segmental duplication | 0.783 | 2.137 | 0.367 | Yes |
| EVM0003977/EVM0004122 | PaCYP6ET1/PaCYP6GF1 | Segmental duplication | 0.612 | 2.063 | 0.297 | Yes |
| EVM0003977/EVM0007979 | PaCYP6ET1/PaCYP420C1 | Segmental duplication | 0.779 | 2.256 | 0.345 | Yes |
| EVM0003977/EVM0009536 | PaCYP6ET1/PaCYP3987C1 | Segmental duplication | 0.883 | 1.853 | 0.476 | Yes |
| EVM0004122/EVM0004208 | PACYP6GF1/PaCYP307A2 | Segmental duplication | 0.918 | 2.602 | 0.353 | Yes |
| EVM0004122/EVM0005751 | PaCYP6GF1/PaCYP6ZZ1 | Segmental duplication | 0.631 | 1.537 | 0.411 | Yes |
| EVM0004122/EVM0007552 | PaCYP6GF1/PaCYP315A1 | Segmental duplication | 1.051 | 2.588 | 0.406 | Yes |
| EVM0004122/EVM0007561 | PaCYP6GF1/PaCYP6PQ2 | Segmental duplication | 0.789 | 3.371 | 0.234 | Yes |

| | | | | | | |
|---|---|---|---|---|---|---|
| EVM0004122/EVM0007575 | PaCYP6GF1/PaCYP325BP1 | Segmental duplication | 0.915 | 3.106 | 0.295 | Yes |
| EVM0004122/EVM0007735 | PaCYP6GF1/PaCYP3997A4 | Segmental duplication | 0.934 | 2.042 | 0.457 | Yes |
| EVM0004208/EVM0005113 | PaCYP307A2/PaCYP3987D1 | Segmental duplication | 1.069 | 1.751 | 0.610 | Yes |
| EVM0004208/EVM0009779 | PaCYP307A2/PaCYP18A1 | Segmental duplication | 0.884 | 2.299 | 0.384 | Yes |
| EVM0004611/EVM0009779 | PaCYP4ZB1/PaCYP18A1 | Segmental duplication | 0.987 | 2.123 | 0.465 | Yes |
| EVM0005550/EVM0007218 | PaCYP4G304/PaCYP4YX2 | Segmental duplication | 0.775 | 2.819 | 0.275 | Yes |
| EVM0005550/EVM0007552 | PaCYP4G304/PaCYP315A1 | Segmental duplication | 0.961 | 1.983 | 0.485 | Yes |
| EVM0005788/EVM0011023 | PaCYP3998B1/PaCYP314A1 | Segmental duplication | 0.969 | 2.406 | 0.403 | Yes |
| EVM0005888/EVM0010956 | PaCYP301A1/PaCYP4YW1 | Segmental duplication | 0.912 | 2.279 | 0.400 | Yes |
| EVM0007561/EVM0011861 | PaCYP6PQ2/PaCYP6EU7 | Segmental duplication | 0.731 | 2.473 | 0.296 | Yes |
| EVM0007735/EVM0009536 | PaCYP3997A4/PaCYP3987C1 | Segmental duplication | 0.750 | 1.740 | 0.431 | Yes |
| EVM0007945/EVM0008668 | PaCYP303A1/PaCYP6GC1 | Segmental duplication | 1.019 | 1.972 | 0.517 | Yes |
| EVM0010028/EVM0010725 | PaCYP6EU8/PaCYP3987A5 | Segmental duplication | 0.964 | 1.622 | 0.594 | Yes |
| EVM0010725/EVM0011861 | PaCYP3987A5/ PaCYP6EU7 | Segmental duplication | 0.913 | 1.930 | 0.473 | Yes |
| EVM0001303/EVM0008669 | PaCYP4G305/PaCYP4G306 | tandem duplication | 0.257 | 1.051 | 0.244 | Yes |
| EVM0006546/EVM0007218 | PaCYP4YX1/PaCYP4YX2 | tandem duplication | 0.154 | 0.280 | 0.549 | Yes |
| EVM0007072/EVM0010725 | PaCYP4702A1/PaCYP3987A5 | tandem duplication | 0.835 | 1.546 | 0.540 | Yes |
| EVM0007328/EVM0011060 | PaCYP4YW2/PaCYP12AZ1 | tandem duplication | 0.925 | 2.147 | 0.431 | Yes |
| EVM0008066/EVM0009599 | PaCYP4D106/PaCYP4D105 | tandem duplication | 0.549 | 1.951 | 0.281 | Yes |
| EVM0008145/EVM0010649 | PaCYP9HL1/PaCYP9AT13 | tandem duplication | 0.484 | 2.960 | 0.164 | Yes |
| EVM0008668/EVM0009272 | PaCYP6GC1/PaCYP6ZY1 | tandem duplication | 0.488 | 1.664 | 0.294 | Yes |
| EVM0001723/EVM0003603 | PaCYP305A34/PaCYP15A1 | tandem duplication | 0.672 | 2.368 | 0.284 | Yes |
| EVM0005770/EVM0007293 | PaCYP9HJ1 PaCYP9HJ2 | tandem duplication | 0.367 | NA | NA | Yes |
| EVM0007469/EVM0007575 | PaCYP325BP1 PaCYP3013A1 | tandem duplication | 0.524 | NA | NA | Yes |

Table S4. The significant change of gut expressed-PaP450 genes. The remarkable alterations are marked in bold.

| Gene Name | Gene ID | CV-5 µg/l group vs CV-50 µg/l group | | | GD-5 µg/l group vs GD-50 µg/l group | | | GDC vs CVC | | |
|---|---|---|---|---|---|---|---|---|---|---|
| | | FDR | log2FC | Regulation | FDR | log2FC | Regulation | FDR | log2FC | Regulation |
| PaCYP12P1v1 | EVM0000161 | 0.999757619 | -0.025613621 | normal | 0.011934384 | -0.655902676 | normal | 0.89521516 | -0.048369782 | normal |
| PaCYP9HK1 | EVM0000292 | 0.999757619 | -0.172958098 | normal | 0.006347362 | -0.80566209 | normal | 0.306965242 | -0.366540088 | normal |
| PaCYP6AAB1 | EVM0000320 | 0.999757619 | 0.431490211 | normal | 0.543798829 | -0.467976689 | normal | 0.946486981 | -0.045017188 | normal |
| PaCYP302A1 | EVM0000338 | -- | -- | -- | -- | -- | -- | -- | -- | -- |
| PaCYP307B1 | EVM0000618 | -- | -- | -- | -- | -- | -- | -- | -- | -- |
| PaCYP4MR6 | EVM0001233 | 0.740227418 | -0.677138195 | normal | 2.11E-07 | -1.76266812 | down | 0.138944537 | 0.413469744 | normal |
| PaCYP4G305 | EVM0001303 | -- | -- | -- | -- | -- | -- | -- | -- | -- |
| PaCYP3997A5 | EVM0001371 | -- | -- | -- | -- | -- | -- | -- | -- | -- |
| PaCYP6FW1 | EVM0001503 | 0.999757619 | -0.653180007 | normal | 1.05E-18 | -3.098304149 | down | 0.331681087 | -0.299403605 | normal |
| PaCYP305A34 | EVM0001723 | 0.999757619 | 0.087737809 | normal | 0.477542538 | -0.385871998 | normal | 0.553884124 | 0.268678126 | normal |
| PaCYP4YZ1 | EVM0001842 | 0.999757619 | 0.202684758 | normal | 0.000872741 | -0.950407082 | normal | 1.81E-06 | 0.35879947 | normal |
| PaCYP6AAA1P | EVM0002438 | 0.999757619 | -0.529235833 | normal | 0.007344122 | -1.680422055 | **down** | 0.095436526 | 0.74433786 | normal |
| PaCYP4ZA1 | EVM0003351 | 0.936271828 | -0.568385515 | normal | 0.000937078 | -1.471210574 | **down** | 0.986751896 | -0.008542417 | normal |
| PaCYP12P2P | EVM0003565 | 0.999757619 | 0.130521638 | normal | 0.219225071 | -0.320355316 | normal | 0.711837787 | 0.174108293 | normal |
| PaCYP15A1 | EVM0003603 | -- | -- | -- | -- | -- | -- | -- | -- | -- |
| PaCYP4C167 | EVM0003657 | 0.999757619 | 0.465936986 | normal | 0.026449106 | 1.320700891 | normal | 0.767426327 | 0.181571164 | normal |
| PaCYP4030A3 | EVM0003672 | 0.999757619 | -0.096454345 | normal | 0.477195339 | -0.468211478 | normal | 0.800500337 | 0.146976384 | normal |
| PaCYP6FX5 | EVM0003931 | 0.999757619 | -0.189289604 | normal | 7.54E-15 | -2.977503988 | **down** | 0.786672823 | 0.106985528 | normal |
| PaCYP6ET1 | EVM0003977 | 0.999757619 | -0.009116339 | normal | 0.001510159 | -0.65036985 | normal | 0.805746347 | 0.073598059 | normal |

| | | | | | | | | | | |
|---|---|---|---|---|---|---|---|---|---|---|
| PaCYP6GF1 | EVM0004122 | 0.999757619 | 0.313404451 | normal | 0.838205647 | 0.043992303 | normal | 5.41E-07 | -0.613794879 | normal |
| PaCYP307A2 | EVM0004208 | -- | -- | -- | -- | -- | -- | -- | -- | -- |
| PaCYP4ZB1 | EVM0004611 | -- | -- | -- | -- | -- | -- | -- | -- | -- |
| PaCYP12P1v2 | EVM0004993 | 0.999757619 | -0.159499865 | normal | 1.36E-08 | -0.619892274 | normal | 0.623974805 | 0.178178506 | normal |
| PaCYP4D107 | EVM0005110 | 0.999757619 | -0.602081389 | normal | 0.371853783 | -0.549083414 | normal | 0.783823507 | -0.132756377 | normal |
| PaCYP3987D1 | EVM0005113 | 0.907128911 | 0.756154822 | normal | -- | -- | -- | -- | -- | -- |
| PaCYP4038B1 | EVM0005123 | 0.999757619 | 0.510743255 | normal | 0.545967351 | 0.096698271 | normal | 0.001163491 | 0.3724822 | normal |
| PaCYP6AAD1 | EVM0005172 | 0.999757619 | -0.034936887 | normal | 2.30E-07 | -1.009409008 | **down** | 0.620281109 | -0.264704949 | normal |
| PaCYP4G304 | EVM0005550 | -- | -- | -- | -- | -- | -- | -- | -- | -- |
| PaCYP3996A5 | EVM0005667 | 0.999757619 | 0.157435005 | normal | 0.055942179 | -0.774457482 | normal | 0.704242451 | 0.147327668 | normal |
| PaCYP6ZZ1 | EVM0005751 | 0.999757619 | -0.136453185 | normal | 0.107950316 | -0.446568548 | normal | 0.374011006 | 0.272070001 | normal |
| PaCYP9HJ1 | EVM0005770 | 0.582950021 | -0.690814625 | normal | 0.341298856 | -0.32541362 | normal | 0.000131038 | -0.246711699 | normal |
| PaCYP3998B1 | EVM0005788 | 0.999757619 | -0.415705263 | normal | 7.97E-05 | -1.734197782 | **down** | 0.118463731 | -0.699689356 | normal |
| PaCYP301A1 | EVM0005888 | 0.999757619 | -0.112422838 | normal | 1.12E-11 | 1.698931114 | **up** | 0.53706575 | -0.338432926 | normal |
| PaCYP4038C1 | EVM0006012 | -- | -- | -- | -- | -- | -- | -- | -- | -- |
| PaCYP4YX1 | EVM0006546 | -- | -- | -- | -- | -- | -- | -- | -- | -- |
| PaCYP6PQ1 | EVM0006654 | 0.999757619 | -0.276715154 | normal | 8.16E-07 | -0.835296278 | normal | 0.967543832 | -0.017025192 | normal |
| PaCYP4702A1 | EVM0007072 | -- | -- | -- | -- | -- | -- | -- | -- | -- |
| PaCYP4703A1 | EVM0007161 | 0.999757619 | 0.4619486 | normal | 0.053128093 | 1.193347522 | normal | 0.696471701 | 0.251081019 | normal |
| PaCYP4YX2 | EVM0007218 | -- | -- | -- | -- | -- | -- | -- | -- | -- |
| PaCYP9HJ2 | EVM0007293 | 0.999757619 | -0.592394923 | normal | 0.017070341 | -0.572181367 | normal | 0.038350039 | -0.698633915 | normal |
| PaCYP4YW2 | EVM0007328 | -- | -- | -- | -- | -- | -- | 0.658285013 | -0.275165837 | normal |
| PaCYP3013A1 | EVM0007469 | -- | -- | -- | -- | -- | -- | 0.743498572 | -0.226313332 | normal |
| PaCYP315A1 | EVM0007552 | 0.999757619 | -0.374029022 | normal | 0.560831467 | 0.348570153 | normal | 0.977157348 | 0.018496425 | normal |
| PaCYP6PQ2 | EVM0007561 | 0.286885531 | -0.984167157 | normal | 0.030519979 | -0.830855324 | normal | 0.484248739 | -0.345538754 | normal |
| PaCYP325BP1 | EVM0007575 | 0.999757619 | -0.628518795 | normal | 5.88E-24 | -2.624279818 | **down** | 3.59E-06 | 0.869926491 | normal |

| | | | | | | | | | | |
|---|---|---|---|---|---|---|---|---|---|---|
| PaCYP3997A4 | EVM0007735 | -- | -- | -- | -- | -- | -- | -- | -- | -- |
| PaCYP303A1 | EVM0007945 | -- | -- | -- | -- | -- | -- | 0.996509563 | 0.003095283 | normal |
| PaCYP420C1 | EVM0007979 | 0.999757619 | 0.188919349 | normal | 1.03E-06 | -2.026296576 | **down** | 0.997521486 | 0.00138415 | normal |
| PaCYP4D106 | EVM0008066 | 0.999757619 | -0.698696426 | normal | 2.44E-09 | -1.998421925 | **down** | 0.06694779 | 0.714165477 | normal |
| PaCYP9HL1 | EVM0008145 | 0.999757619 | 0.301024284 | normal | 0.033820008 | -0.565535174 | normal | 0.694992802 | -0.166714474 | normal |
| PaCYP6GC1 | EVM0008668 | 0.999757619 | -0.103173863 | normal | 0.002177869 | -0.927934371 | normal | 0.007214216 | 0.484139386 | normal |
| PaCYP4G306 | EVM0008669 | -- | -- | -- | -- | -- | -- | -- | -- | -- |
| PaCYP306A1 | EVM0008860 | 0.999757619 | -0.029991102 | normal | 0.343704146 | 0.331792428 | normal | 0.77549344 | 0.09473434 | normal |
| PaCYP6ZY1 | EVM0009272 | 0.999757619 | 0.325172251 | normal | 0.063857581 | -0.721974418 | normal | 0.937165744 | -0.046052662 | normal |
| PaCYP3987C1 | EVM0009536 | -- | -- | -- | -- | -- | -- | -- | -- | -- |
| PaCYP4D105 | EVM0009599 | 0.999757619 | -0.301663194 | normal | 1.72E-05 | -1.440754401 | **down** | 0.75400455 | 0.167669442 | normal |
| PaCYP18A1 | EVM0009779 | 0.999757619 | 0.359952845 | normal | 0.013356582 | 1.634478349 | normal | 0.764478216 | 0.161494625 | normal |
| PaCYP6EU8 | EVM0010028 | 0.69760679 | -0.804062596 | normal | 8.46E-10 | -1.615243487 | **down** | 0.65538807 | -0.280107441 | normal |
| PaCYP9AT13 | EVM0010649 | 0.999757619 | 0.074777717 | normal | 0.63771133 | -0.084107319 | normal | 0.048310833 | -0.402221728 | normal |
| PaCYP3987A5 | EVM0010725 | -- | -- | -- | -- | -- | -- | -- | -- | -- |
| PaCYP4YW1 | EVM0010956 | 0.999757619 | 0.099290054 | normal | 0.065201856 | 0.599217369 | normal | 0.472032749 | -0.289075981 | normal |
| PaCYP314A1 | EVM0011023 | 0.999757619 | -0.665974861 | normal | 2.58E-06 | -1.907657615 | **down** | 0.139881919 | -0.603007984 | normal |
| PaCYP12AZ1 | EVM0011060 | 0.999757619 | -0.201196265 | normal | 0.001091199 | -0.350497583 | normal | 0.910883488 | -0.037880747 | normal |
| PaCYP6AAC1 | EVM0011176 | 0.999757619 | -0.025613621 | normal | -- | -- | -- | -- | -- | -- |
| PaCYP6EU7 | EVM0011861 | 0.999757619 | -0.172958098 | normal | 5.19E-05 | -1.497877914 | **down** | 0.963132994 | -0.01920504 | normal |

Table S5. The comparison of gut expressed-PaP450 genes between CV and GD samples. The remarkable alterations are marked in bold.

| Gene Name | Gene ID | GN-5 µg/l group vs CV-5 µg/l group | | | GD-5 µg/l group vs GD-50 µg/l group | | |
|---|---|---|---|---|---|---|---|
| | | FDR | log2FC | Regulation | FDR | log2FC | Regulation |

| | | | | | | | |
|---|---|---|---|---|---|---|---|
| PaCYP6FX5 | EVM0003931 | 0.000436208 | 1.954359266 | **up** | 0.274394604 | -0.488382927 | normal |
| PaCYP325BP1 | EVM0007575 | 5.47E-22 | 3.103531481 | **up** | 7.11E-06 | 1.293248468 | **up** |
| PaCYP420C1 | EVM0007979 | 0.007190843 | 1.583188755 | **up** | 0.023155389 | -0.560089454 | normal |
| PaCYP4YZ1 | EVM0001842 | 2.10E-05 | 1.503162329 | **up** | 0.176643433 | 0.366596953 | normal |
| PaCYP6FW1 | EVM0001503 | 0.001144378 | 1.517680403 | **up** | 0.433413411 | -0.392306712 | normal |
| PaCYP301A1 | EVM0005888 | 0.007327191 | -1.143497409 | **down** | 0.042152501 | 0.616073789 | normal |
| PaCYP3987D1 | EVM0005113 | 0.315647891 | 0.201567804 | normal | 0.001965708 | -1.104092689 | **down** |
| PaCYP3998B1 | EVM0005788 | 0.880779455 | 0.111265203 | normal | 0.005186411 | -1.050572294 | **down** |
